# Learning to Classify DWDM Optical Channels from Tiny and Imbalanced Data

**DOI:** 10.3390/e23111504

**Published:** 2021-11-13

**Authors:** Paweł Cichosz, Stanisław Kozdrowski, Sławomir Sujecki

**Affiliations:** 1 Computer Science Institute, Warsaw University of Technology, Nowowiejska 15/19, 00-665 Warsaw, Poland; p.cichosz@elka.pw.edu.pl; 2 Faculty of Electronics, Military University of Technology, S. Kaliskiego 2, 00-908 Warsaw, Poland; slawomir.sujecki@wat.edu.pl; 3 Telecommunications and Teleinformatics Department, Wroclaw University of Science and Technology, Wyb. Wyspianskiego 27, 50-370 Wroclaw, Poland

**Keywords:** machine learning, optical networks, imbalanced data, one-class classification

## Abstract

Applying machine learning algorithms for assessing the transmission quality in optical networks is associated with substantial challenges. Datasets that could provide training instances tend to be small and heavily imbalanced. This requires applying imbalanced compensation techniques when using binary classification algorithms, but it also makes one-class classification, learning only from instances of the majority class, a noteworthy alternative. This work examines the utility of both these approaches using a real dataset from a Dense Wavelength Division Multiplexing network operator, gathered through the network control plane. The dataset is indeed of a very small size and contains very few examples of “bad” paths that do not deliver the required level of transmission quality. Two binary classification algorithms, random forest and extreme gradient boosting, are used in combination with two imbalance handling methods, instance weighting and synthetic minority class instance generation. Their predictive performance is compared with that of four one-class classification algorithms: One-class SVM, one-class naive Bayes classifier, isolation forest, and maximum entropy modeling. The one-class approach turns out to be clearly superior, particularly with respect to the level of classification precision, making it possible to obtain more practically useful models.

## 1. Introduction

Constantly growing traffic in backbone networks makes dynamic and programmable optical networks increasingly important. This particularly applies to Dense Wavelength Division Multiplexing (DWDM) networks whereby efficient use of network resources is of paramount importance. Introducing automation, frequent network reconfiguration, re-optimization and network reliability monitoring allows DWDM network operators to minimize the capital expenditures (Capex) and operating expenditures (Opex) [1,2,3,4,5,6]. Currently, software-defined networking (SDN) is used to achieve all these objectives. SDN uses a logically centralized control plane in a DWDM network that is realized using purpose-built flexible hardware such as reconfigurable optical add/drop multiplexers (ROADMs), flexible line interfaces, etc. [7,8]. In modern DWDM optical networks, following the software defined network paradigm, DWDM network reconfiguration is becoming more frequent, making the evolving network more resilient and adapting faster to real changes in bandwidth demand so that network reconfigurations can closely match changes in bandwidth demand. However, bandwidth demand can change very quickly (fluctuations can occur within minutes), while network reconfigurations typically take much longer. This is mainly due to operational processes that are too slow to allow real-time network re-optimization. It is therefore important that DWDM network reconfigurations are automated and as fast as possible, without significantly increasing operational costs.

Frequent network reconfiguration and re-optimization necessary to make the best use of available resources has been facilitated by the introduction of software-defined networking (SDN) and knowledge-based networking (KDN) paradigms [7,9,10,11]. Central to SDN and KDN is automatic provisioning of optical channels (lightpaths), which is based on accurate quality estimation for them. Machine learning (ML) is a promising solution to this problem. Therefore, a number of algorithms have been proposed that first create a database using numerical modelling tools and then implement ML to estimate the quality of optical links ([6,7,12]). However, in the approach presented here, we apply ML to a database that has been extracted directly via the control plane from the DWDM network under analysis. This approach leads to an ML problem that is clearly different from the one addressed in [6,7,11,12], as there are significant challenges in using real optical network datasets, related to data representation, data size and class imbalance, which is intrinsic to data gathered via control plane from an operating DWDM network. The class imbalance follows from the fact that in an operating DWDM network there may be dozens or hundreds of operating connections but there is not much information available (if any) on connections that could not be realized due to excessive bit error rate. Therefore, a specially tailored ML approach is required to tackle this problem.

The main advantage of gathering data via the control plane is that it can be easily implemented by a DWDM network operator. As it will be further explained later, this approach imposes some constraints on the choice of appropriate ML methods due to above-mentioned class imbalance, which is an intrinsic feature of data collected via control plane. As already mentioned this makes the ML problem considered in this contribution clearly different from those considered so far in most of the available literature [6,7,12,13]. Expanding upon our previous work [14,15], we compare the predictive performance of the most successful binary classification algorithms combined with different techniques for class imbalance compensation and that of one-class classification algorithms that learn from majority class instances only.

### 1.1. Machine Learning Challenges

Successful applications of machine learning to support optical network design require real training data. While experiments on synthetic data may provide encouraging demonstrations, they are likely not to adequately represent the challenges that are associated with this application area and therefore provide overoptimistic predictive performance estimates or fail to identify potential obstacles and culprits. These challenges are mainly related to data size and quality.

DWDM network operators, particularly operating small or medium networks, may be unable to provide a dataset with more than several dozen or at best several hundred paths. More importantly, the vast majority if not all of those path configurations would usually correspond to correct, working channel designs. This is because unsuccessful path configurations are often discarded rather than archived, at least before the provider becomes aware of their utility as training data for machine learning. Before this awareness increases, available real datasets remain tiny and extremely imbalanced.

The data that has been made available for this study comes from a DWDM network operator providing services in Poland and is an excellent example of these issues. It contains just about a hundred of paths, including only three “bad” ones (i.e., such that could not be allocated due to a low quality of transmission). While it is still possible to use such data to train predictive models using classification learning algorithms, special care is needed to increase their sensitivity to the minority class and to reliably evaluate their quality. The extreme dominance of the “good” class makes it easy to come up with apparently accurate models with little or no actual predictive utility. To avoid this, we compensate the class imbalance using instance weighting and synthetic minority-class instance generation. However, the tiny size and extreme imbalance of the data may be still on the edge of the capabilities of standard binary classification, even with such compensation techniques. Therefore one-class classification, in which only “good” paths are used as training data, may be a viable and promising alternative. To compare the predictive performance of the binary and one-class classification approaches, their predictions are evaluated using ROC and precision-recall curves in combination with stratified cross-validation.

### 1.2. Related Work

To the authors’ best knowledge this work is the first to apply one-class classification in the optical network domain and to compare its predictive performance to binary classification using different techniques of handling class imbalance. There is, however, some related prior work on applying other machine learning methods to optical networks as well as on using one-class classification as an alternative to binary classification for imbalanced data.

Considering work related to optical networks first, in [16] authors show that a routing and spectrum allocation (RSA) that monitors QoT in multiple slices significantly improves network performance. Rottondi et al. [12] extensively discuss and use ML techniques to optimise complex systems where analytical models fail. However, the network data in [12] was generated artificially, whereas in this contribution the data is collected by control plane from an operating network.

Similar problems related to lightpath QoT estimation are addressed by Mata et al. [17] but they mainly focus on the SVM classifier only. Barletta et al. [18] on the other hand, use mainly Random Forest algorithm that predicts whether the BER (Bit Error Rate) of unestablished lightpaths meets the required threshold based on traffic volume, desired route and modulation format. As in [12] the system is trained and tested on artificial data, which is different to the approach adopted in this contribution.

Japkowicz [19] compared different ways of handling class imbalance including one-class classification. Japkowicz [19] found binary classification with imbalanced compensation superior to one class classification but experiments performed in [19] used artificial data and neural network classifier (with one-class classification performed using an autoassociative network type).

Lee and Cho [20] advocated the use of one-class classification for imbalanced data and demonstrated that it can outperform binary classification if the imbalanced ratio is high. They experimented with the standard and one-class versions of the SVM algorithm.

Bellinger et al. [21] discuss the potential utility of one-class classification in binary classification tasks with extreme class imbalance, as in our case. Their results suggest that binary classification with class imbalance compensation methods may be more useful than one-class classification when dealing with data from complex multi-modal distributions. However their results are based on datasets where the number of minority class instances is bigger than in our case.

### 1.3. Article organization

The rest of the paper is organized as follows. In Section 2 the analyzed optical network data, the applied machine learning algorithms, and model evaluation methods are described. The results of the experimental study are presented in Section 3 and discussed in Section 4. Contributions of this work and future research directions are summarized in Section 5.

## 2. Materials and Methods

The data comes from a real DWDM optical network of a large telecom operator. The network uses 96 DWDM channels allocated in C-band and is physically located in Poland, with network nodes corresponding to Polish cities.

### 2.1. Data

The network is equipped exclusively with coherent transponders. This is a typical representative of a new network created by an operator. The coherent transponders belong to Ciena’s 6500 family, with transmission rates of 100 G, 200 G and 400 G and four types of modulation: QPSK, 16QAM, 32QAM and 64QAM.

**Figure 1:**
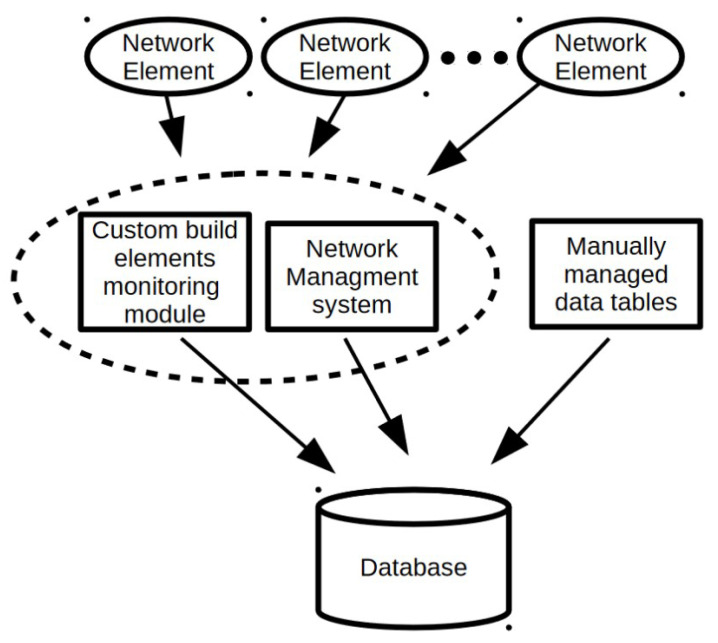
Data preparation process.

**Figure 2:**
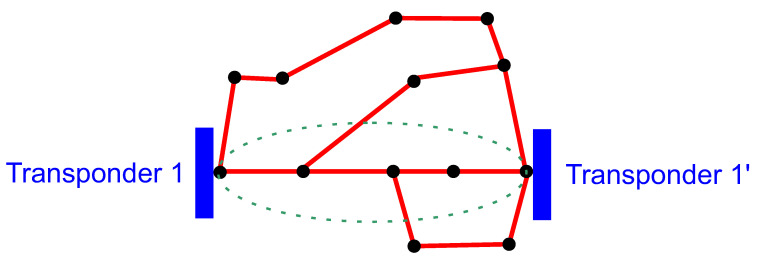
An example DWDM network topology.

**Figure 3:**
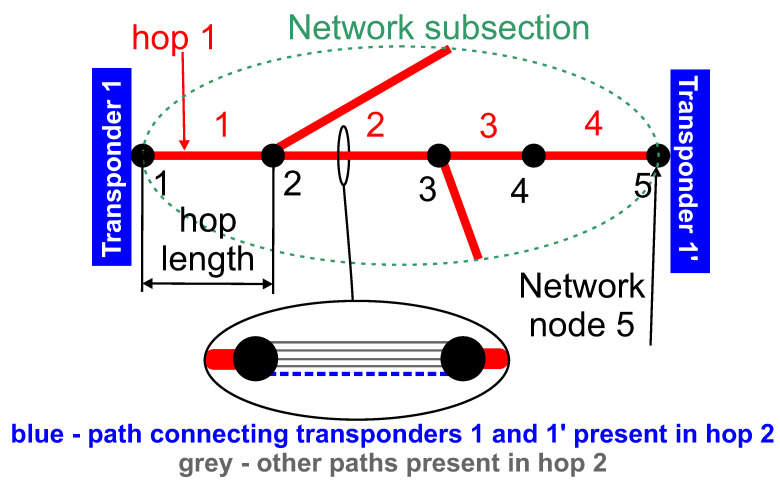
Network subsection illustrating the meaning of the specific channel attributes occurring in the studied database.

Data preparation process is depicted in Figure 1. In order to better understand the meaning of the various database attributes presented later in the subsection, in the context of DWDM technology, an example DWDM network topology is shown in Figure 2. Figure 3 illustrates the concepts of network node, hop, hop length, path, and transponder. The dataset contains 107 optical paths, 3 of which correspond to unsuccessful designs (“bad”) and rest of them (104) are operational (“good”).

#### 2.1.1. Path Description

Network paths are described by several properties that may be related to transmission quality and expected to be predictively useful. The hop_lengths property gives the length of each hop that forms a path from the initial transponder to the destination transponder. This property is important because the signal to noise ratio depends on the length of the fibre connecting both transponders. In each hop there are usually more wavelengths occupied. This is because these wavelengths are used by paths other than the one occupied by the considered path. All paths can interact through nonlinear phenomena like four wave mixing and thus affect the quality of transmission. Therefore, the num_of_paths_in_hops property, which gives the number of adjacent DWDM wavelengths in a given hop, is included. The hop_losses property gives the value of the optical loss for a given hop. Again, hop losses affect the signal to noise ratio and hence the corresponding property was included. Another property, number_of_hops, provides information on how many hops are present in a path from the initial to destination transponder. Since each hop corresponds to a signal passing through a DWDM node, the number of hops affects the signal to noise ratio due to optical regeneration taking place in a DWDM node. The last two properties are intrinsically related with a specific type of transponder used. The transponder_modulation property stores information on the transponder modulation format, e.g., QPSK or 16QAM. This property is important because modulation format is related to receiver sensitivity. Finally, the transponder_bitrate property is in essence self explanatory and gives the bit rate of a given transponder. Transponder bit rate also affects receiver sensitivity and hence it is included.

#### 2.1.2. Vector Representation

Path descriptions were transformed to a vector representation, as expected by classification algorithms for tabular data, by a simple aggregation-based feature engineering technique. Each of the available edge properties (hop_lengths, num_of_paths_in_hops, hop_losses) was aggregated by calculating the mean and standard deviation over all edges in the path. This gives 6 attributes derived from edge properties (2 attributes for each of the 3 edge properties), in addition to the 3 path attributes unrelated to individual edges (number_of_hops, transponder_modulation, and transponder_bitrate).

Applying additional aggregation functions to edge properties, such as the minimum, the maximum, the median, the first quartile, the third quartile, or the linear correlation coefficient with the ordinal number of the edge in the path, as in our prior work [14], may create some additional predictively useful attributes. However, this would make the dimensionality of this representation relatively high in comparison to the size of the available dataset, considerably increasing the risk of overfitting.

### 2.2. Binary Classification

Any standard classification algorithm can be used to predict channel “good”/“bad” class labels or probabilities. In this work we limit our attention to the two algorithms that performed the best in our previous study [14]: Random forest and extreme gradient boosting. They belong to the most successful learning algorithms for tabular data and it is very unlikely that their performance could be beaten by other algorithms using the same vector path representation.

#### 2.2.1. Random Forest

The random forest algorithm creates a model ensemble consisting of multiple decision trees [22]. They are grown on bootstrap samples from the training set by using a mostly standard decision tree growing algorithm [23,24]. However, since the expected improvement of the resulting model ensemble over a single model is contingent upon sufficient diversity of the individual models in the ensemble [25,26], the following modifications are applied to stimulate the diversity of decision trees that are supposed to constitute a random forest:

- large maximally fitted trees are grown (with splitting continued until reaching a uniform class, exhausting the set of instances, or exhausting the set of possible splits),
- whenever a split has to be selected for a tree node, a small subset of available attributes is selected randomly and only those attributes are considered for candidate splits.

To use a random forest model for prediction, simple unweighted voting of individual trees from the model is performed, and vote distribution is used to obtain class probability predictions. With dozens or (more typically) hundreds trees this voting mechanism usually makes random forests highly accurate and resistant to overfitting. An additional important advantage of the algorithm is its ease of use, resulting from limited sensitivity to parameter settings, which makes it possible to obtain high quality models without excessive tuning.

#### 2.2.2. Extreme Gradient Boosting

Extreme gradient boosting or *xgboost* is is another highly successful ensemble modeling algorithm. As other boosting algorithms, it creates ensemble components sequentially in such a way that each subsequent model best combines with the previously created ones [27,28,29,30].

The *xgboost* algorithm internally uses regression trees for model representation and optimizes an ensemble quality measure that includes a loss term and a regularization term [31]. Each subsequent tree is grown to minimize the sum of loss and regularization terms of all trees so far. Split selection criteria, stop criteria, and leaf values are derived from this minimization by the Taylor expansion of the loss function, using its gradient and hessian decomposed to terms for particular training instances and then assigned to the corresponding nodes and leaves of the tree being grown.

Extreme gradient boosting applied to binary classification is typically used with logarithmic loss (the negated log-likelihood) and the summed up numeric predictions of individual regression trees are transformed by a logistic link function to obtain class probability predictions.

The extreme gradient boosting algorithm is capable of providing excellent prediction quality, sometimes outperforming random forest models. It can overfit, however, if the number of trees grown is too large.

#### 2.2.3. Handling Class Imbalance

Techniques for compensating class imbalance can be divided in the following three main categories:

- internal compensation by the learning algorithm, controlled by its parameter settings,
- compensation by data resampling,
- compensation by synthetic minority class data generation.

Techniques of the first category are generally supposed to increase sensitivity to the minority class without modifying the training data. They are possible with many classification algorithms and often consist in specifying class weights or prior probabilities. The binary classification algorithms used by this work are both ensemble modeling algorithms, which tend to be quite robust with respect to class imbalance, but their model quality can be still improved by such compensation mechanisms.

In the case of the random forest algorithm there are actually two possible related techniques. One is drawing bootstrap samples in a stratified manner, with different selection probabilities for particular classes. In the extreme case, a bootstrap sample may contain all instances from the minority class and the sample of the same size from the dominating class. The other is to specify instance weights affecting split selection and stop criteria for tree growing. Since in our case classes are extremely imbalanced and there are very few instances of the minority class, the weighting technique is preferred to the stratified sampling technique, since the latter would have to severely undersample the dominating class, with a possibly negative effect on model performance. The same weighting technique is also used with the the *xgboost* algorithm. In this case instance weights are used when calculating the logarithmic loss, so that the contribution of minority class instances to the loss function minimized by the algorithm is increased.

Data resampling may be performed by minority class oversampling (replicating randomly selected minority class instances), majority class undersampling (selecting a sample of majority class instances), or a combination of both, so that the resampled training set has either fully balanced classes or at least considerably more balanced than originally. Unfortunately these techniques have very limited utility for datasets that are both small and extremely imbalanced, as in our case. Undersampling would remove most of the available training data, and oversampling would replicate the very few “bad” paths increasing the risk of overfitting to these specific instances. They can be therefore hardly expected to offer any advantages over internal imbalance compensation by weighting and are not used in this work.

Potentially more useful techniques of generating synthetic minority class instances can be considered more refined forms of oversampling in which minority class instances available in the training data are not directly replicated, but used to generate new synthetic instances. This is supposed to make the increased representation of the minority class in the modified training set more diverse and thus reduce the risk of overfitting. Two well known specific techniques based of this idea are SMOTE [32] and ROSE [33] and they are both used in our experimental study. SMOTE finds nearest neighbors of each minority class instance and then generates new synthetic instances by interpolating between the instance and its neighbors. ROSE adopts a smoothed bootstrap sampling approach, with new instances generated in the neighborhood of original instances by drawing from a conditional kernel density estimate of attribute values given the class. Both minority and majority class instances are generated, and the class distribution in the generated dataset can be controlled to achieve a desired level of balance.

### 2.3. One-Class Classification

One-class classification follows the following learning scenario [34,35]:

- the training contains only instances of a single class,
- the learned model is supposed to predict for any instance whether it belongs to the single class represented in the training set.

In our case the single class represented in the training set corresponds to “good” paths. When the obtained model is applied to prediction, it identifies paths which are likely to also be “good” (i.e., be of the same class as that represented in the training set) and those which are likely to be “bad” (i.e., not to be of the same class as that represented in the training set). It can be assumed that model predictions are provided in the form of decision function values (numeric scores) such that higher values are assigned to instances that are considered less likely to be if the same class as that represented in the training set, i.e., in our case, more likely to be “bad” paths.

One-class classification is most often applied to unsupervised or semi-supervised anomaly detection [36], where an unlabeled training set, assumed to contain only normal instances, sometimes with a small fraction of anomalous instances, is used to learn a model that can detect anomalous instances in new data. It can be also useful, however, for binary classification tasks with extreme class imbalance [21], particularly when the number of minority class instances is too small for standard binary classification algorithms, even combined with imbalance compensation techniques. This may be often the case with data for optical channel classification.

The best known and widely used one-class classification algorithm is one-class SVM. In our experimental study it is compared with three other algorithms: The one-class naive Bayes classifier, the isolation forest algorithm, and the maximum entropy modeling algorithm. The first of those is a straightforward modification of the standard naive Bayes classifier and probably the simplest potentially useful one-class learning algorithm. The second one, while designed specifically for anomaly detection applications, can also serve as a general-purpose one-class classification algorithm. The third one, while originally intended for creating models of species distribution in ecosystems, has been also found to be useful for one-class classification.

#### 2.3.1. One-Class SVM

The one-class SVM algorithm uses a linear decision boundary, like standard SVM, but adopts a different principle to determine its parameters. Rather than maximizing the classification margin, which is not applicable to one-class classification, it maximizes the distance from the origin while separating the majority of training instances therefrom [37]. The side of the decision boundary opposite from the origin corresponds to the class represented in the training set. Only a small set of outlying training instances are permitted to be left behind, and the upper bound on the share of such outlying instances in the training set is specified via the ν parameter.

The principle of separating most of the training set from the origin of the space is typically combined with a translation-invariant kernel function (such as the radial kernel), sothat instances in the transformed representation lie on a sphere centered in the origin. The separating hyperplane then cuts off a segment of the sphere where most training instances are located.

One-class SVM predictions are signed distances from the decision boundary, positive on the “one-class” (normal) side and negative on the outlying side. The negated value of such signed distance can therefore serve as a numeric score for ranking new instances with respect to their likelihood of not belonging to the class represented in the training set.

#### 2.3.2. One-Class Naive Bayes Classifier

The one-class modification of the naive Bayes classifier is particularly straightforward. Since only one class is represented in the training set, its prior probability is assumed to be 1, conditional attribute-value probabilities within this class are estimated on the full training set, and the probability of an instance belonging to this class is proportional to the product of such attribute-value probabilities [38]. For numeric attributes, Gaussian density function values, with the mean and standard deviation estimated on the training data, are used instead of discrete attribute-value probabilities.

Discrete class predictions can be made by comparing the product of attribute-value probabilities for a given instance to a threshold, set to or around the minimum value of this product over the training set. Numeric scores (decision function values) can be defined as the difference between such a threshold and the probability being compared.

#### 2.3.3. Isolation Forest

The isolation forest algorithm was proposed as an anomaly detection method [39], but it can also serve as a one-class classification algorithm regardless of whether instances not belonging to the class represented in the training set are interpreted as anomalous. Its model representation consists of multiple isolation trees grown with random split selection. These are not standard decision or regression trees, since no labels or values are assigned to leaves, and they just partition the input space. Splitting is stopped whenever a single training instance is left or a specified maximum depth is reached.

In the prediction phase each isolation tree is used to determine the path length between the root node and the leaf at which the instance arrives after traversing down the tree along splits. Instances that do not belong to the class represented in the training set can be expected to be easier to isolate (have shorter paths) than those which do belong to the class. The average path length over all trees in the forest can then serve as a decision value function for determining whether an instance is likely to belong to this class or not. The original algorithm transforms this average path length into a standardized anomaly score for generating alerts in anomaly detection applications, using the expected depth of unsuccessful BST searches. This is not necessary for one-class classification, since the negated average path length is sufficient to rank new instances with respect to their likelihood of not belonging to the class represented by the training set.

An extended version of the isolation forest used for this work employs multivariate rather than univariate splits [40]. This eliminates a bias that resulted in the original algorithm from using axis-parallel hyperplanes for data splitting.

#### 2.3.4. Maximum Entropy Modeling

The maximum entropy modeling or maxent algorithm was originally developed for ecological species geographical distribution prediction based on available presence data, i.e., locations where a given species has been found and their attributes, used to derive environmental features [41]. These features, besides raw continuous attribute values, include attributes derived by several transformations, as well as binary features obtained by comparing continuous attributes with threshold values and by one-hot encoding of discrete attributes, with an internal forward feature selection process employed based on nested model comparison [42].

The algorithm, following the maximum entropy principle [43], identifies a species occurrence probability distribution that has a maximum entropy (i.e., is most spread out) while preserving constraints on environmental features. These constraints require that the expected values of environmental features under the estimated species occurrence probability distribution should be close to their averages from the presence points. The obtained model can provide, for an arbitrary point, the prediction of the species occurrence probability.

Despite its original intended purpose, maxent has been found to be useful as a general-purpose one-class classification algorithm [44,45]. Training instances take the role of “presence points”, and input attributes are used to derive “environmental features”, whereas background points can be generated by uniformly sampling the attribute ranges. Model prediction for an arbitrary instance can be interpreted as the probability that it belongs to the class represented in the training set.

### 2.4. Model Evaluation

Both binary and one-class classification algorithms used by this work produce scoring predictive models – their predictions are numeric values ranking instances with respect to the likelihood of being a “bad” path (or not belonging to the class represented in the training data). When applying standard binary classification quality measures to evaluate these predictions using, we refer to to the “good” class (represented in the training data for one-class classification), as *negative*, and the “bad” class (not represented in the training data for one-class classification) as *positive*.

ROC and precision-recall (PR) curves are used to visualize the predictive performance of the obtained models. ROC curves make it possible to observe possible tradeoff points between the *true positive rate* (the share of positive instances correctly predicted to be positive) and the *false positive rate* (the share of negative instances incorrectly predicted to be positive) [46,47]. PR curves similarly present possible levels of tradeoff between the *precision* (the share of positive class predictions that are correct) and the *recall* (the same as the true positive rate). The overall predictive power is summarized using the area under the ROC curve (AUC) and the area under the precision-recall curves (PR AUC).

In our case the true positive rate and the recall is the share of “bad” paths that are correctly predicted to be “bad”, the false positive rate is the share of “good” paths that are incorrectly predicted to be “bad”, and the precision is the share of “bad” class predictions that are correct. The area under the ROC curve can be interpreted as the probability that a randomly selected “bad” path is scored higher by the model than a randomly selected “good” path and the area under the PR curve can be interpreted as the average precision across all recall values.

When using data with heavily imbalanced classes, where positive instances are extremely scarce, even numerous false positives do not substantially decrease the false positive rate, since the number of false positives may be still small relative to the dominating negative class count. This is not the case for the precision, though, which is much more sensitive to false positives. Therefore precision-recall curves may be expected to be more informative and better highlight differences in the predictive performance obtained using different algorithms. For a more complete picture, however, both ROC and PR curves are presented.

To make an effective use of the small available dataset for both model creation and evaluation as well as to keep the evaluation bias and variance at a minimum, the n×k-fold cross-validation procedure (n×k-CV) is applied [48]. The dataset is split into *k* equally sized subsets, each of which serves as a test set for evaluating the model created on the combined remaining subsets, and this process is repeated *n* times to further reduce the variance. To evaluated binary classification models, the random partitioning into *k* subsets is performed by stratified sampling, preserving roughly the same number of minority class instances in each subset. For the evaluation of one-class classification models a one-class version of the n×k-CV procedure is used in which the few instances of the “bad” class are never used for training but always used for testing. The true class labels and predictions for all n×k iterations are then combined to determine ROC curves, PR curves, and the corresponding AUC values.

While it appears a common practice to use the leave-out-out procedure rather than *k*-fold cross-validation when working with small datasets, the only potential advantage of the former would be avoiding the pessimistic bias resulting from the fact that in each iteration of the latter $\frac{1}{k}$ of the data are not used for model creation. However, the leave-one-out procedure has high variance (that cannot be reduced by multiple repetitions since the procedure is fully deterministic) and excluding only a single instance from the training data may cause optimistic bias due to underrepresenting the differences between the training data and the test data. We find it therefore more justified to use the n×k-fold cross-validation procedure where the variance is substantially reduced and accept the fact that it may be pessimistically biased. This means that our reported results may underestimate the actually possible predictive performance levels, which should be preferred to any risk of optimistic bias.

## 3. Results

In the experimental study presented in this section binary and one-class classification algorithms described in Section 2.2 and Section 2.3 are applied to the small and imbalanced dataset described in Section 2.1. The objective of the study is to verify the level of optical channel classification quality that can be obtained using these two types of algorithms. For binary classification the effects of class imbalance compensation using instance weights and synthetic minority class instance generation are also examined.

### 3.1. Algorithm Implementations and Setup

The following algorithm implementations are used in the experiments:

- **random forest (RF):** the implementation provided by the ranger R package [49],
- **extreme gradient boosting (XGB):** The implementation provided by the xgboost R package [50],
- **SMOTE:** The implementation provided by the smotefamily R package [51],
- **ROSE:** The implementation provided by the ROSE R package [52],
- **one-class SVM (OCSVM):** The implementation provided by the e1071 R package [53],
- **one-class naive Bayes classifier (OCNB):** The implementation provided by the e1071 R package [53], with a custom prediction method to handle the one-class classification mode specifically implemented for this work,
- **isolation forest (IF):** The implementation provided by the isotree R package [54],
- **maximum entropy modeling (ME):** The implementation provided by the MIAmaxent R package [55], with background data generation by random sampling of attribute value ranges specifically implemented for this work.

Since the *xgboost* algorithm does not directly support discrete attributes and one attribute in the dataset is discrete, it was preprocessed by converting discrete values to binary indicator columns.

The tiny size of the dataset and, particularly, the number of “bad” path configurations makes it hardly possible to perform algorithm hyper-parameter tuning. While the performance evaluation obtained by n×k-fold cross-validation could be used to adjust algorithm settings and improve the results, as demonstrated in our previous work [14], without the possibility to evaluate the expected predictive performance of the tuned configurations on new data it could lead to overoptimistic results. This is why the algorithms are used in the following mostly default configurations, with only a few parameters set manually where defaults are unavailable or clearly inadequate:

- **random forest:** A forest of 500 trees is grown, with the number of attributes drawn at random for split selection set to the square root of the number of all available attributes,
- **extreme gradient boosting:** 50 boosting iterations are performed, growing trees with a maximum depth of 6 and scaling the contribution of each tree by a learning rate factor of 0.3, and applying $L_{2}$ regularization on leaf values with a regularization coefficient of 1,
- **SMOTE:** The number of nearest neighbors of minority class instances is set to 1 (which is the only available choice given the fact there are just three minority class instances in the data two of which are available for model creation in each cross-validation fold),
- **ROSE:** The generated dataset size and the probability of the minority class are set so as to approximately preserve the number of majority class instances and increase the number of minority class instances,
- **one-class SVM:** The radial kernel function is used, with the γ kernel parameter set to the reciprocal of the input dimensionality, and the ν parameter specifying an upper bound on the share of training instances that may be considered outlying is equal 0.5,
- **isolation forest:** The extended version of the algorithm is used [40], with multivariate splits based on three attributes, a forest of 500 isolation trees is grown, and for each of them the data sample size is equal the training set size (which is a reasonable setup for a small dataset), and the maximum three depth is the ceiling of the base-2 logarithm thereof,
- **maximum entropy modeling:** All available attribute transformations [42] are applied to derive environmental features (linear, monotone, deviation, forward hinge, reverse hinge, threshold, and binary one-hot encoding), a significance threshold used for internal feature selection is set to 0.001, and the generated background data size is 1000.

For imbalance compensation with instance weighting the majority class weight is fixed as 1 and the minority class weight is set to values from the following sequence: 1,2,5,10,20,50,100 (where 1 corresponds to no weighting). When using synthetic instance generation, the number of generated minority class instances is set to d−1 times the number of real minority class instances, where *d* is in the same sequence as above. This can be achieved exactly for SMOTE and only approximately for ROSE due to its probabilistic nature.

The n×k-fold cross-validation procedure is used with k=3 (since there are only 3 minority class instances) and n=50 (to keep the evaluation variance at a minimum).

### 3.2. Classification Performance

For each of the binary and one-class classification algorithm configurations described above cross-validated ROC and PR curves, with the corresponding area under the curve values, are reported and briefly discussed below. A bootstrap test (with 2000 replicates drawn from the data) is used for verifying the statistical significance of the observed AUC differences.

#### 3.2.1. Binary Classification

**Figure 4:**
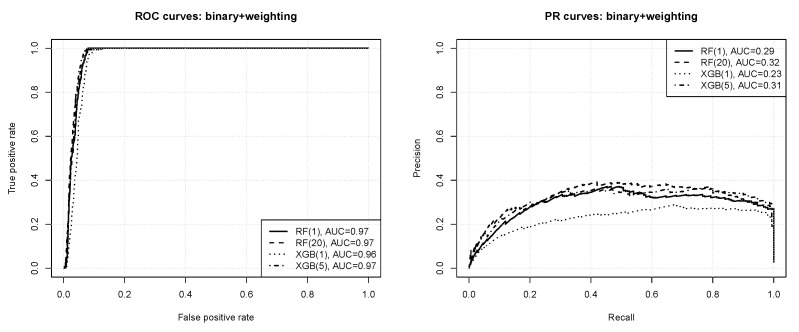
The ROC and PR curves for binary classification with instance weighting.

Figure 4 presents the ROC and PR curves obtained for binary classification with instance weighting. The numbers in the parentheses after algorithm acronyms in the plot legends specify the minority instance weight value. For readability, only the results without weighting and with the best weight value are included. All the observed differences are statistically significant according to the bootstrap test except for those between RF(1) and XGB(5), and between RF(20) and XGB(5). One can observe that:

- according to the ROC curves the prediction quality appears very good, with AUC values of 0.96–0.97,
- nearly perfect ROC operating points are possible, with the true positive rate of 1 and the false positive rate of 0.05 or less,
- the precision-recall curves reveal that the prediction quality is not actually perfect, with the average precision just above 0.3 at best,
- without instance weighting the random forest algorithm outperforms *xgboost*, but with instance weighting they both perform on roughly the same level,
- imbalance compensation with instance weighting improves the predictive performance of both the algorithms, with the effect more pronounced for extreme gradient boosting.

**Figure 5:**
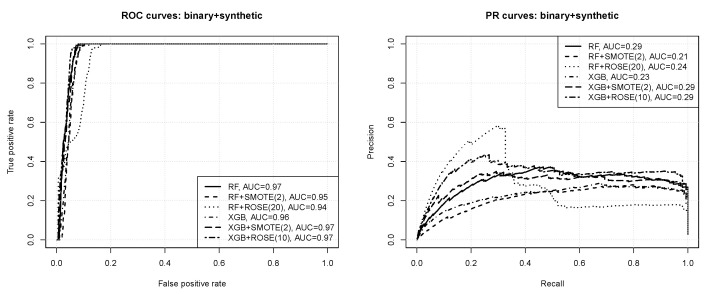
The ROC and PR curves for binary classification with synthetic instance generation.

Figure 5 presents the ROC and PR curves obtained for binary classification with synthetic instance generation. The numbers in the parentheses after algorithm acronyms in the plot legends specify the minority class size multiplication coefficient. For readability, only the best results obtained when using SMOTE and ROSE are included and the results with no synthetic instance generation as a comparison baseline. All the observed differences are statistically significant according to the bootstrap test. One can observe that:

- synthetic instance generation reduces the prediction quality of the random forest algorithm, but provides an improvement for extreme gradient boosting,
- the effects of SMOTE and ROSE for *xgboost* are similar except for the fact that the latter works better with bigger minority class multiplication coefficients,
- the results for both SMOTE and ROSE are worse than those obtained with instance weighting.

#### 3.2.2. One-Class Classification

**Figure 6:**
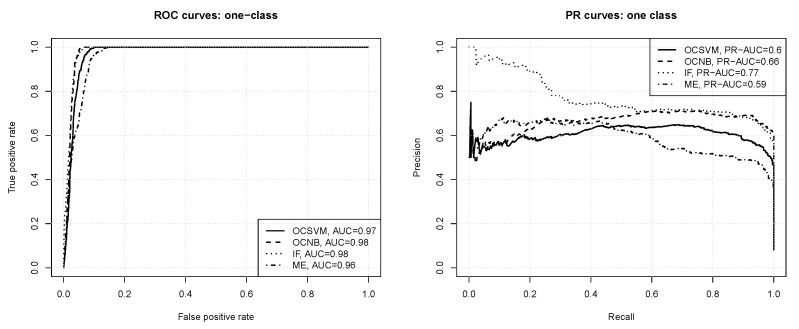
The ROC and PR curves for one-class classification.

The ROC and precision-recall curves for one-class classification are presented in Figure 6. All the observed differences are statistically significant according to the bootstrap test except for the one between IF and OCNB. One can observe that:

- all the algorithms produce models capable of successfully detecting out-of-class instances (“bad” paths), with AUC values between 0.96 and 0.98,
- the one-class naive Bayes and isolation forest algorithms achieve the maximum true positive rate value for a slightly less false positive rate value than the one-class SVM and maxent algorithms,
- the algorithms differ more substantially with respect to the average precision achieved, which is about 0.6 for one-class SVM and maxent, 0.66 for the one-class naive Bayes classifier, and 0.77 for the isolation forest algorithm,
- the isolation forest and one-class naive Bayes models maintain a high precision of 0.7 or above for a wide range of recall values (up to about 0.9), whereas the one-class SVM and maxent models can only maintain a precision level of 0.6 and 0.5, respectively, in the same range of recall values,
- all the one-class algorithms produce better models than those obtained by binary classification.

## 4. Discussion

As discussed in Section 2.4, ROC curves do not provide a sufficient picture of model performance under severe class imbalance, because even with many false positives the false positive rate remains small due to the dominating overall negative class count. This is why they suggest that all the investigated algorithms achieve excellent prediction quality and their models exhibit only minor performance differences. Precision-recall curves indeed show a more useful view of the predictive quality of models obtained by particular algorithms and better highlight the differences between them.

For binary classification algorithms the simple instance weighting technique appears more useful than the more refined and computationally expensive synthetic instance generation techniques. This may be surprising at first, but actually neither SMOTE or ROSE are well suited to working with datasets not only heavily imbalanced but also very small. With just three minority class instances (two remaining for model creation within a single cross-validation fold) there is probably not enough real data to provide a reliable basis for synthetic data generation.

One-class classification algorithms, although using less input information (training data of the majority class only), all produce clearly better models than the best of those obtained using binary classification. The isolation forest algorithm turns out to deliver a superior overall predictive power and considerably more preferable operating points, with near-perfect detection of true positives (“bad” paths) without excessively many false positives. While all the algorithms deliver high quality models, the one-class naive Bayes and isolation forest algorithms clearly outperform the one-class SVM and maxent algorithms. It is particularly noteworthy that they can provide high precision in a wide range of recall values.

This study suggests that standard methods of handling class imbalance may be insufficient when the dataset is of a very small size. Indeed, it is not only the small share, but also the small absolute number of “bad” paths that prevents binary classification algorithms from creating more successful models. While the skewed class distribution can be compensated for by weighting, just a few training instances provide very poor basis for detecting generalizable patterns and for generating synthetic instances. Using only “good” paths for model creation leads to better results. The best obtained one-class models providing a precision level of about 0.7 are much more practically useful than the best binary classification models with precision just above 0.3.

## 5. Conclusions

The work has provided additional evidence that applying machine learning to optical channel classification is a promising work direction, but is associated with important challenges. To achieve models applicable in real-world conditions one has to use real-world datasets, but these suffer from severe imperfections, the most important of which are a small size and a heavy class imbalance. We have demonstrated that state-of-the-art binary classification algorithms may not achieve a very high level of prediction quality even when coupled with appropriate imbalance compensation techniques. The utility of the latter may be limited by the fact that it is not only the relative share of the minority class instances in the data that is small, but also their absolute count. The reported results confirm that one-class classification is a viable alternative, and models learned using majority class data only achieve better classification precision that those obtained using binary classification learning from all data.

Our findings provide an encouragement to continue this research direction by extending input representation with additional attributes, applying more one-class classification algorithms, and tuning their parameters to further improve the predictive performance. Gathering additional data not only would make the results of these enhanced future studies more reliable, but also make it possible to examine further ideas, such as model transfer between different networks or combining models trained on data from different networks. Expert knowledge on the physics of optical networks may permit defining alternative or additional path attributes, creating a more adequate input space representation for machine learning. Such knowledge could also be used to design a domain-specific data augmentation method that might be expected to perform better than general-purpose techniques of synthetic minority-class instance generation.

## Data Availability

Not applicable.
